# Flat band ferromagnetism in Pb$_2$Sb$_2$O$_7$ via a self-doped mechanism

**DOI:** 10.1038/s41598-023-31917-w

**Published:** 2023-03-23

**Authors:** I. Hase, Y. Higashi, H. Eisaki, K. Kawashima

**Affiliations:** 1 National Institute of Advanced Industrial Science and Technology (AIST), Tsukuba Central 2, 1-1-1 Umezono, Tsukuba, 305-8568 Japan; 2 IMRA-JAPAN Material R &D Co. Ltd., 2-1 Asahi-machi, Kariya, Aichi 448-0032 Japan

**Keywords:** Materials science, Physics

## Abstract

Electron systems with strong geometrical frustrations have flat bands, and their unusual band dispersions are expected to induce a wide variety of physical properties. However, for the emergence of such properties, the Fermi level must be pinned within the flat band. In this study, we performed first-principles calculations on pyrochlore oxide Pb$_2$Sb$_2$O$_7$ and theoretically clarified that the self-doping mechanism induces pinning of the Fermi level in the flat band in this system. Therefore, a very high density of states is realized at the Fermi level, and the ferromagnetic state transforms into the ground state via a flat band mechanism, although the system does not contain any magnetic elements. This compound has the potential to serve as a new platform for projecting the properties of flat band systems in the real world.

## Introduction

Systems that exhibit unusual band structures (dispersion relations) have attracted considerable attention in recent years in condensed matter physics. Among these, systems with a dispersion-free band, known as the flat band, are theoretically predicted to exhibit several novel properties, such as perfect ferromagnetism and high-temperature superconductivity, owing to their unique band structures^1–9^. Furthermore, such systems have attracted considerable attention from a topological perspective. Combined with the special band structure, interesting physical properties such as the integer/fractional quantum Hall effect and fractional Chern insulator phase are theoretically expected to appear^10–13^. Recently, a flat band device utilizing these special properties has also been proposed^14^.

A flat band appears when isotropic hopping occurs between nearest neighbor lattice points in a lattice model with strong geometrical frustration. In other words, the band dispersion vanishes owing to the quantum destructive interference of the wavefunction resulting from strong geometric frustration. Numerous lattices with geometric frustrations have been described in Ref.^5^. Among these, the most extensively studied is the kagome lattice for two-dimensional (2D) systems and the pyrochlore lattice for three-dimensional (3D) systems. This is because these lattices can achieve flat bands without requiring special conditions for inter-site hopping.

Numerous attempts have been made to realize the flat band model in real-world systems. In 2D systems, quantum wire networks^15^, photonic crystals^16,17^, laser cooling of atoms^18^, metal–organic frameworks (MOFs)^19^, and twisted bilayer graphene^20^ belong to this category. However, few attempts have been made to develop 3D systems. Pyrochlore A$_2$B$_2$O$_7$ (A = Sn, Pb, Tl; B = Nb, Ta)^21,22^ and WN$_2$^23^ have recently been developed as 3D systems with nearly flat bands; however, the ferromagnetic behavior predicted by first-principles calculations is yet to be confirmed experimentally. This could be attributed to several factors, but the most critical one is the difficulty of carrier doping into the flat bands.

The concept of geometric frustration has also been applied to localized spin systems to explain several unusual magnetic properties. In numerous pyrochlore oxides, magnetic elements with localized spins are arranged on a pyrochlore lattice, and the geometrical frustration of the lattice creates unusual physical properties, such as quantum spin liquids, spin ice, and magnetic monopoles^24–28^. Lattices with strong geometric frustration are more generally defined as line graphs on bipartite lattices^2^, and kagome and checkerboard lattices, as well as pyrochlore lattices, fall in this category. Previous studies have also suggested that magnetic ions form a kagome lattice in Herbertsmithite ZnCu$_3$(OH)$_6$Cl$_2$ and possess the properties of a quantum spin liquid^29–32^. Evidence supporting quantum spin liquids is also found in KCu$_6$AlBiO$_4$(SO$_4$)$_5$Cl, which has a geometrically frustrated square-kagome lattice structure^33^.

Flat bands also appear in localized spin systems. Magnon excitations in frustrated spin systems have flat magnon bands, and the ground state near the saturation field can be understood as the crystalline state of localized magnon excitations^5,6^. Because the interaction between the two localized spins is isotropic and short range in most cases (e.g., the Heisenberg model), the appearance of a flat band is expected. However, for itinerant systems, this situation is more complicated. Interorbital hopping is isotropic if the related orbitals are *s* orbitals; however, for other orbitals (for example, *p*, *d*, or *f* orbitals), interorbital hopping is not necessarily isotropic. Therefore, there exist only few compounds in which flat bands appear in itinerant systems. Exceptionally, the *s* orbitals of the A site in pyrochlore A$_2$B$_2$O$_7$ (A = Sn, Pb, Tl; B = Nb, Ta)^21,22^ and the *p* orbitals of the N atom in WN$_2$^34^ form quasi-flat bands near the Fermi level. Note that none of these are normally magnetic orbitals, such as *d* or *f* orbitals. Nevertheless, first-principles calculations predict that these compounds have a ferromagnetic ground state owing to the large density of states (DOS) derived from the flat band if they are doped appropriately.

Pb$_2$Sb$_2$O$_7$ is popular for its use in ancient Egypt as a yellow pigment, called Naples yellow. Some studies have reported that it has a pyrochlore structure^34,35^, whereas other studies have reported that it has a weberite structure^36–38^. The structure of the produced compound also differs depending on the synthesis conditions. Recently, a first-principles calculation of Pb$_2$Sb$_2$O$_7$ was reported^34^. Pb$_2$Sb$_2$O$_7$ can be synthesized using NaCl flux to produce a compound partially containing Na^34^. The authors attributed the lack of formation of Na-free Pb$_2$Sb$_2$O$_7$ in this environment to the presence of “highly localized states near the Fermi level”. In Na-free Pb$_2$Sb$_2$O$_7$, as indicated in Ref.^34^, there exist “highly localized states near the Fermi level,” which represents the “holy grail” for our analysis, i.e., a naturally carrier-doped flat band. Very recently, a paper titled “Catalogue of flat band stoichiometric materials” was published; here, Pb$_2$Sb$_2$O$_7$ was briefly introduced as an example of a flat band material^39^.

In this study, we demonstrate that pyrochlore oxide Pb$_2$Sb$_2$O$_7$ exhibits ferromagnetism without carrier doping by first-principles calculations and that similar to Sn$_2$Nb$_2$O$_7$, this system has a flat band (FB1) mainly consisting of Pb-*s* orbitals at the top of the valence band (VBM). The most striking feature of this system is the overlap of another flat band (hereafter referred to as FB2), which mainly consists of Sb-*s* orbitals. This causes some of the electrons in FB1 to flow into FB2, resulting in the hole doping of FB1.

The remainder of this paper is organized as follows. The calculation methods and results are described in “Methods and results” section. Conclusions are given in “Conclusions” section.

## Methods and results

We computed the electronic structure of Pb$_2$Sb$_2$O$_7$ from first-principles calculations based on the density functional theory^40,41^. Details on these calculations can be found in Supplemental Material for details of the calculation, which includes Refs.^40–42^.

**Figure 1:**
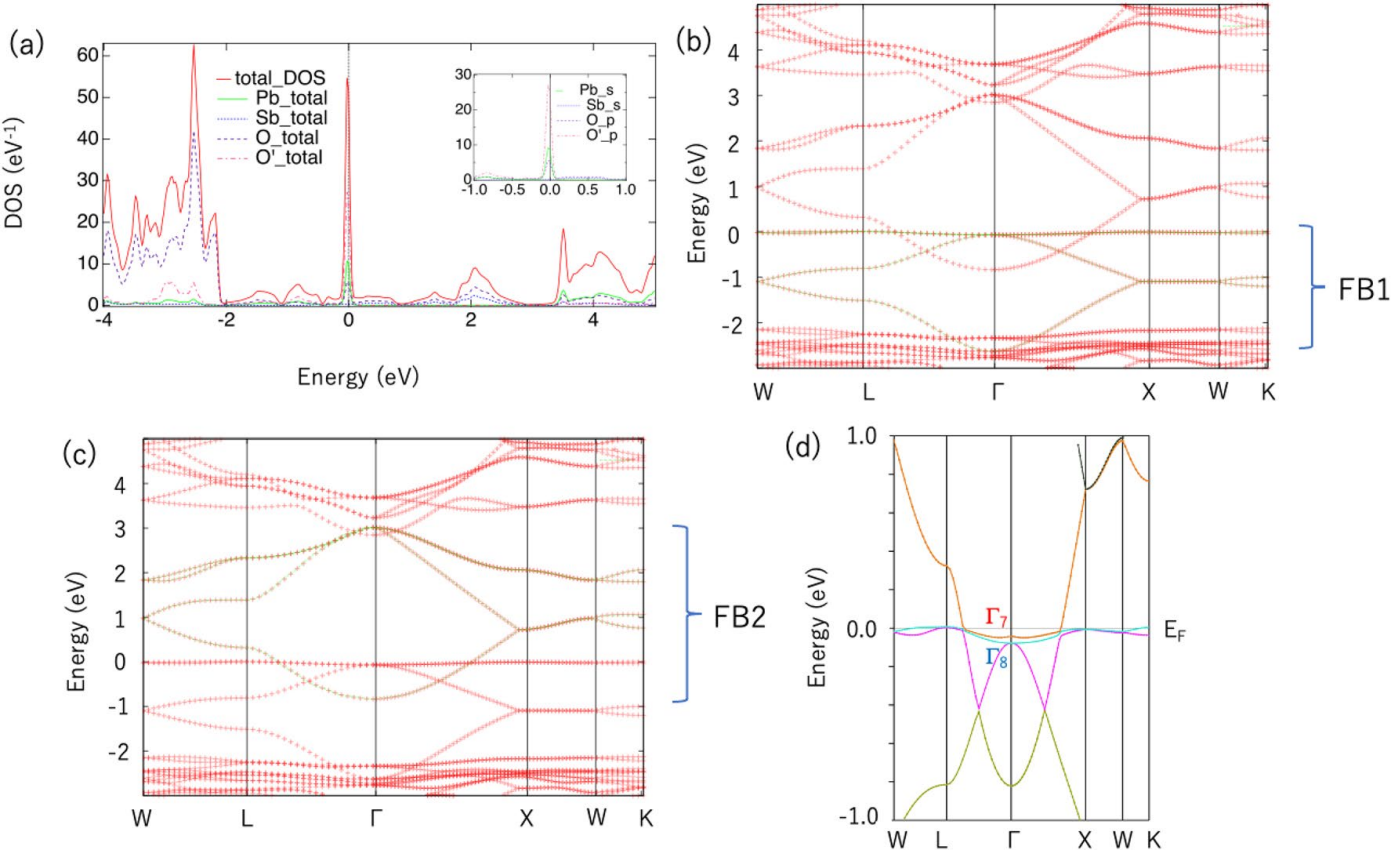
(**a**) DOS curve of Pb$_2$Sb$_2$O$_7$ from first-principles calculations. The inset shows a zoomed-in view of the DOS, including the Pb-*s*, Sb-*s*, O-*p*, and O$^{\prime }$-*p* components. (**b**) Energy dispersion of Pb$_2$Sb$_2$O$_7$. Red points are obtained from our first-principles calculations, and green points (FB1) are obtained from maximally localized Wannier function (MLWF) fitting with four “Pb-*s*” orbitals. (**c**) Red points are the same as in (**b**), and green points (FB2) are obtained by MLWF fitting with four “Sb-*s*” orbitals. (**d**) Energy dispersion of Pb$_2$Sb$_2$O$_7$ near $E_\text{F}$, including spin-orbit interaction. $E_\text{F}$ is set to 0 eV or the VBM. The parameters used in (**b,c**) are listed in Table 1 and in Supplemental Material for details of the calculation, which includes Refs.^40–42^.

Figure 1a presents the DOS curves of Pb$_2$Sb$_2$O$_7$ in the nonmagnetic state. A clear flat band can be observed at the Fermi level ($E_\text{F}$). This flat band has a similar origin to the flat band in Sn$_2$Nb$_2$O$_7$; that is, it is mainly composed of *s* orbitals of Pb atoms located in the pyrochlore lattice and *p* orbitals of O atoms (O$^{\prime }$ sites) surrounded by Pb atoms.

We can construct a four-orbital tight-binding model with four *s* orbitals centered at Pb atoms (“Pb-*s*” orbitals) in the primitive unit cell (PUC) of Pb$_2$Sb$_2$O$_7$ using the MLWF^42^ (see Supplemental Material for details of the calculation, which includes Refs.^40–42^). The original valence band can be perfectly reproduced, as shown in Fig. 1b. The four largest parameters are listed in Table 1. The number of bands can be easily counted along the X–Z–W axis because all bands are doubly degenerate on this axis owing to the non-symmorphic space group. Similarly, the conduction bands can be reproduced using four MLWFs centered at Sb atoms (“Sb-*s*” orbitals) in the same PUC of Pb$_2$Sb$_2$O$_7$. The results are depicted in Fig. 1c. When we ignore parameters other than the first-nearest hopping $t_1$, these bands convert into two perfect flat bands. Therefore, we refer to these bands as FB1 and FB2, respectively. We can clearly observe that the top of FB1 is nearly flat, whereas the top of FB2 is significantly bent.

The first two parameters $\epsilon$ and $t_1$ determine the position and total bandwidth of FB ($=W$), respectively. The bandwidth of the highest band of FB (= $W_\text{FB}$) is determined by other parameters, such as $t_2$ and $t_3$. Because the bandwidth of FB1 is extremely narrow, the next-nearest neighbor hopping parameter $t_2$ is very small: $t_2=0.025$ eV. Further, $t_3$ is also smaller in Pb$_2$Sb$_2$O$_7$ than in Sn$_2$Nb$_2$O$_7$. For FB2, $t_2$ and $t_3$ have larger absolute values than those in FB1. This corresponds to a larger $W_\text{FB}$ in FB2.

However, contrary to the expectation of the ionic configuration Pb$^{+2}_2$Sb$^{+5}_2$O$^{-2}_7$, Pb$_2$Sb$_2$O$_7$ becomes metallic. This is because the energy of the Sb-*s* orbital forming the conduction band is lower than that of the Nb-*d* orbital in the conduction band of Sn$_2$Nb$_2$O$_7$; thus, the energy gap collapses.

**Table 1:** Tight-binding parameters for pyrochlore Pb$_2$Sb$_2$O$_7$ and Sn$_2$Nb$_2$O$_7$.

	Pb$_2$Sb$_2$O$_7$				Sn$_2$Nb$_2$O$_7$	
	FB1		FB2		FB	
	$\epsilon ,t$	Distance (*a*)	$\epsilon ,t$	Distance (*a*)	$\epsilon ,t$	Distance (*a*)
$\epsilon$	− 0.586	On-site	1.539	On-site	− 0.7037	On-site
$t_1$	− 0.266	0.3536	− 0.344	0.3536	− 0.2764	0.3536
$t_2$	− 0.025	0.7071	− 0.049	0.6124	− 0.037	0.7071
$t_3$	− 0.009	1.0607	− 0.043	0.7071	0.013	0.6124

(a) Four-orbital MLWF model centered with the Pb site for FB1 of Pb$_2$Sb$_2$O$_7$, (b) four-orbital MLWF model centered with the Sb site for FB2 of Pb$_2$Sb$_2$O$_7$, and (c) four-orbital MLWF model centered with the Sn site for the flat band of Sn$_2$Nb$_2$O$_7$. Details on the notation are presented in Supplemental Material for details of the calculation, which includes Refs.^40–42^. All energy units are electron volt. The distance is expressed in units of *a*, the lattice constant of each compound.

**Figure 2:**
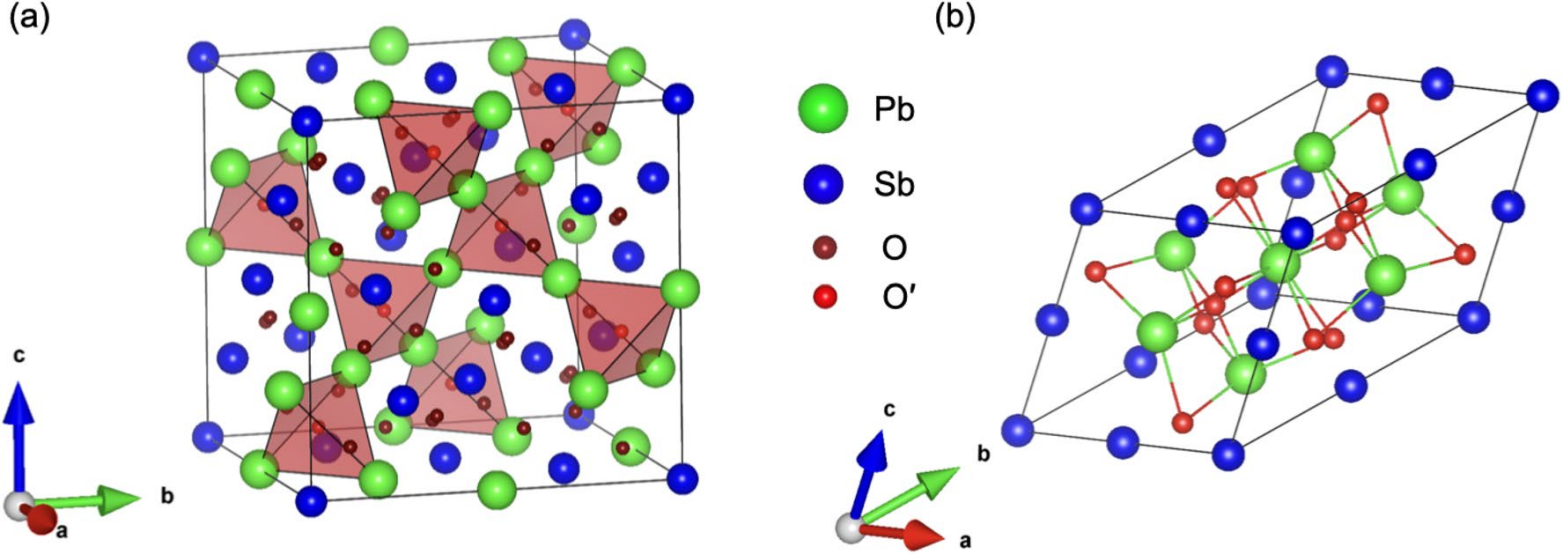
Crystal structure of pyrochlore Pb$_2$Sb$_2$O$_7$ for the (**a**) conventional unit cell and (**b**) PUC. A 3D corner-sharing network of Pb$_4$O$^{\prime }$ tetrahedra is highlighted. The illustration of the crystal structures was produced using VESTA^43^. The internal atomic positions in the PUC were generated using cif2cell^44^.

This behavior can be explained by considering the crystal structure of Pb$_2$Sb$_2$O$_7$, which is shown with a cubic pyrochlore structure in Fig. 2. Pyrochlore oxide A$_2$B$_2$O$_7$ can also be expressed as A$_2$B$_2$O$_6$O$^{\prime }$ owing to the presence of two oxygen sites. A$_2$B$_2$O$_6$O$^{\prime }$ can be decomposed into a corner-shared A$_4$O$^{\prime }$ tetrahedral network (A$_2$O$^{\prime }$ unit) and corner-shared BO$_6$ octahedral network (B$_2$O$_6$ unit)^21^. Let us consider only the A-*s* and B-*s* orbitals. In this case, the pyrochlore lattices of the A and B atoms form separate flat bands (FB1 and FB2, respectively). For the A$_2$O$^{\prime }$ unit, the inclusion of O$^{\prime }$-*p* orbitals does not affect FB1 because of symmetry^21^. Conversely, for the B$_2$O$_6$ unit, the inclusion of O-*p* orbitals severely affects FB2, and the topmost band of FB2 is significantly bent.

The flatness of FB1 is described by the ratio of the width of the entire FB1 band (*W*) to that of the flat band ($W_\text{FB}$) at the top of FB1, that is, $r = W_\text{FB}/W$. This ratio is an important parameter that represents the strength of the property as a flat band. The value of *r* is approximately 0.1 for Sn$_2$Nb$_2$O$_7$ and 0.025 for Pb$_2$Sb$_2$O$_7$. Note that this *r* value is even lower than the *r* value of 0.043 for a typical 2D MOF^45^.

Because the flat band system may cause interesting topological behavior owing to spin-orbit interaction (SOI)^6^, we also performed a band calculation with SOI (see Supplemental Material for details of the calculation, which includes Refs.^40–42^). Figure 1d presents the energy dispersion of Pb$_2$Sb$_2$O$_7$ near $E_\text{F}$ including SOI. Without SOI, the quasi-flat band touches the dispersive band at the $\Gamma$ point^46,47^. In Pb$_2$Sb$_2$O$_7$, the state at this point is specified by the threefold (sixfold if spin is included) degenerated $\Gamma _{25}$ irreducible representation without SOI. When SOI is included, this state splits into twofold $\Gamma _7$ and fourfold $\Gamma _8$ states, as shown in Fig. 1d, for Pb$_2$Sb$_2$O$_7$.

Because this energy splitting is sufficiently small with respect to the bandwidth of FB1 (= *W*), we can apply the Guo and Franz model, which describes a 3D topological insulator on a pyrochlore lattice^47^. The spin-orbit coupling parameter $\lambda$ is described as $\lambda = \Delta E/24$, where $\Delta E = E(\Gamma _7) - E(\Gamma _8)$ denotes the energy splitting at the $\Gamma$ point. In Pb$_2$Sb$_2$O$_7$, $\Delta E = 0.0347$ eV is positive, which follows a general trend for A$_2$B$_2$O$_7$ (A = Tl, Pb; B = Nb, Ta)^22,48^.

The high DOS in $E_\text{F}$ (= high $D(E_\text{F})$) destabilizes the system and induces various phase transitions; the most typical is the phase transition to ferromagnetism. In particular, previous studies have rigorously proved that slight on-site Coulomb repulsion *U* changes the ferromagnetic state to the ground state in an ideal flat band system^1,4^. Even in a system with quasi-flat bands (owing to electron hopping other than to the nearest neighbor site), a numerical study has demonstrated that a ferromagnetic ground state is realized under a sufficiently large *U*^49^. The criterion for *U* to separate the ferromagnetic and paramagnetic ground states is $U \sim W_\text{FB}$, where $W_\text{FB}$ represents the width of the quasi-flat band. Due to the expanded nature of Wannier orbitals, quantitatively estimating *U* on “Pb-*s*” Wannier orbitals is not an easy task^50^. However, in this case, it is plausible to expect *U* to be larger than $W_\text{FB} \sim 0.07$ eV. We also obtain the exact result wherein the ferromagnetic ground state is stable to small flat band bending^51^.

**Figure 3:**
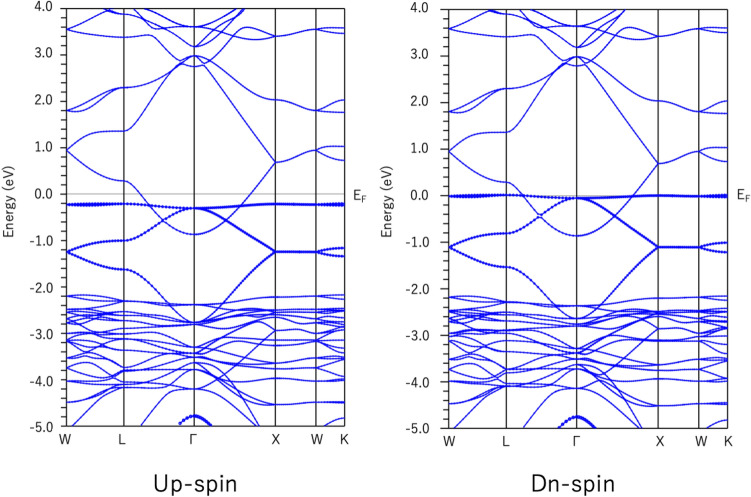
Energy dispersion of ferromagnetic Pb$_2$Sb$_2$O$_7$ for up-spin and down-spin.

Because pyrochlore Pb$_2$Sb$_2$O$_7$ is already metallic, carriers need not be doped to achieve a ferromagnetic state. Further, spin-polarized first-principles calculations were performed. The band structures of the spin-up and spin-down states are shown in Fig. 3. An exchange splitting of approximately 0.2 eV occurs between the up-spin and down-spin bands. FB1 is completely occupied by the up-spin band but partially occupied by the down-spin band. That is, $E_\text{F}$ is pinned at the topmost band of FB1 for the down-spin band. The total magnetic moment is 0.307 $\mu _\text{B}$ per Pb$_2$Sb$_2$O$_7$. The ground state is not half metallic, unlike doped Sn$_2$Nb$_2$O$_7$, because FB2 is nearly equally occupied with up- and down-spins.

Next, we discuss the phase stability of pyrochlore Pb$_2$Sb$_2$O$_7$. As presented above, a large $D(E_\text{F})$ value may induce instability. We have already demonstrated that a ferromagnetic transition lowers the total energy; however, structural phase transitions may also occur. Furthermore, a previous report has suggested that the pyrochlore phase may be unstable^36^. Therefore, we also computed the electronic structure of weberite Pb$_2$Sb$_2$O$_7$ and compared the total energies of the pyrochlore phase (P-phase) and weberite phase (W-phase). At this time, the calculation is performed ensuring that the calculation conditions are almost identical. Consequently, the W-phase is more stable by approximately 0.549 eV per Pb$_2$Sb$_2$O$_7$. Because the ferromagnetic stabilization energy in the P-phase is approximately 0.014 eV, we can conclude that the W-phase is more stable than the P-phase under normal conditions. The W-phase is an insulator with a bandgap of $\Delta = 2.08$ eV. This result is consistent with the experimental observation that Naples yellow is yellow in color. For the W-phase, we did not find any feature of a flat band in the energy dispersion (see Supplemental Material for details of the calculation, which includes Refs.^40–42^).

Another report suggested that Pb$_2$Sb$_2$O$_7$ adopts a pyrochlore structure but is rhombohedrally distorted^35^. In this regard, the band structure of Tl$_2$Nb$_2$O$_7$ with a similar small strain is computed^52^; however, the flat band itself is preserved only by changing the fine structure. Therefore, Pb$_2$Sb$_2$O$_7$ may retain the flat band, even if a small distortion is applied.

**Figure 4:**
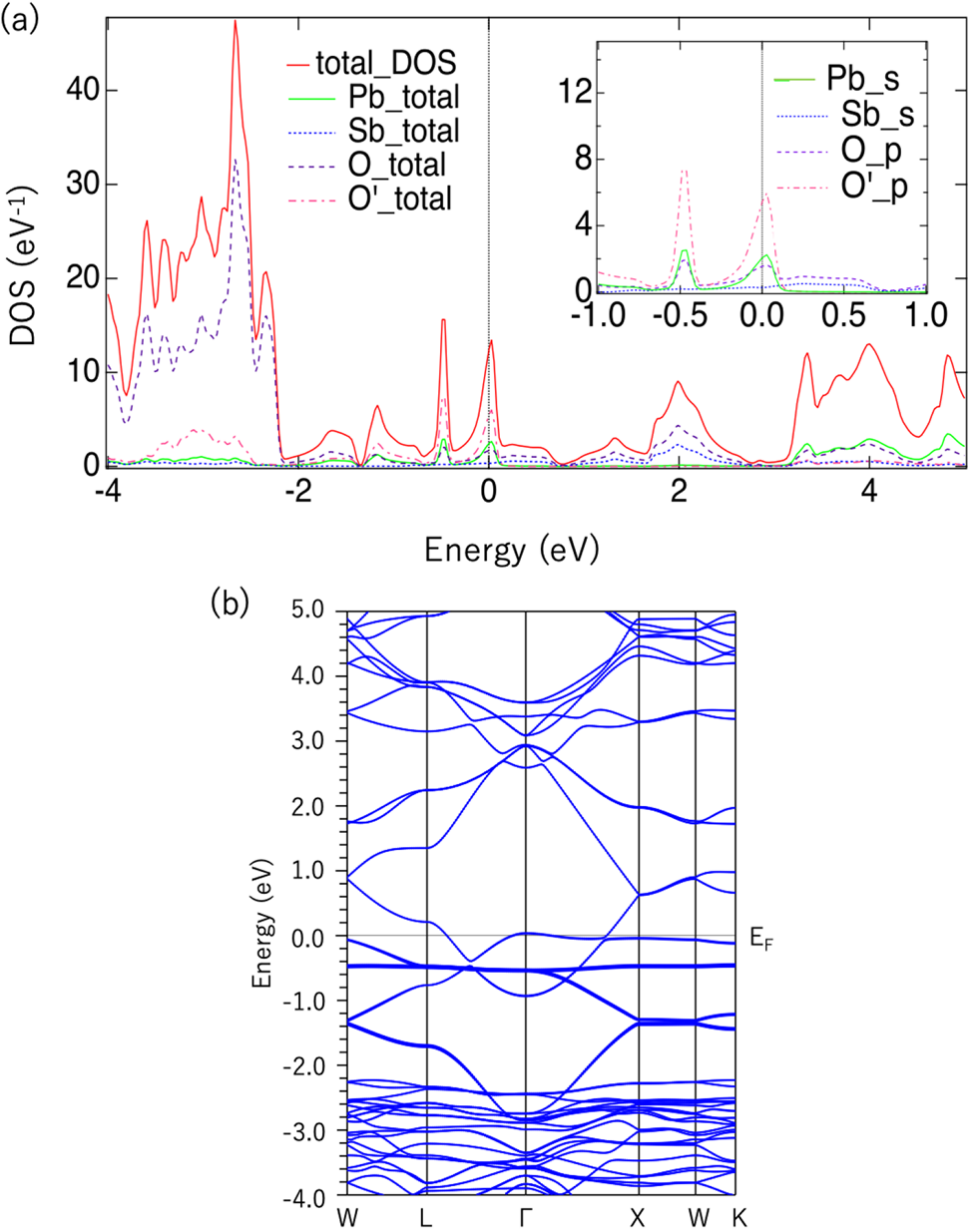
(**a**) DOS and (**b**) energy dispersion of Pb$_2$Sb$_2$O$_7$ with rhombohedral distortion.

To confirm this point, we performed a first-principles calculation with an imposed rhombohedral distortion. Details of the calculations are described in Supplemental Material for details of the calculation, which includes Refs.^40–42^; however, note that we relaxed both crystal lattice and internal atomic positions. We concluded that the loss of symmetry displaced the oxygen atoms considerably, and the electronic state changed accordingly. Figure 4 shows the DOS and band dispersion at the optimized atomic positions. The DOS peak near $E_\text{F}$, which was singular in the cubic crystal, is split into two. The band dispersion is also partially split for the FB. Despite the flat band splitting, we found that $D(E_\text{F})$ was sufficiently high to accept ferromagnetic transition. The spin-polarized calculation showed that the total magnetic moment was 0.37 $\mu _B$ per Pb$_2$Sb$_2$O$_7$, which is higher than that in the cubic phase. This result shows the robustness of the flat band against the rhombohedral distortion.

Rhombohedral distortion may lead to another interesting phenomenon: charge disproportionation due to splitting in two of the single Sb sites in the cubic crystal of the rhombohedral crystal. For example, in BaSbO$_3$, the valence of Sb is Sb$^{4+}$ in the high symmetry phase, but when the symmetry decreases, it splits into two types: Sb$^{3+}$ and Sb$^{5+}$. In BaSbO$_3$-based superconductors, it has been suggested that this charge fluctuation may assist superconductivity^53^. To verify this, we calculated the density at the first radial mesh point (RTOxx in WIEN2k) of a given atom as an approximation for the electron density within the nucleus^54^. We obtained the value $\rho _0$(Sb2)/$\rho _0$(Sb1) = 1.002 for Pb$_2$Sb$_2$O$_7$. This is much smaller than the ratio of 1.55 for BaSbO$_3$ and 1.32 for BaBiO$_3$^53^, which means that almost no charge disproportionation occurs in Pb$_2$Sb$_2$O$_7$. The reason for this is unclear at this stage, but we suspect that the possible Fermi surface nesting in the (111) direction is imperfect in Pb$_2$Sb$_2$O$_7$. However, in BaSbO$_3$, the entire Fermi surface can disappear by folding the Brillouin zone from the Pm$\bar{3}$m structure to the Fm$\bar{3}$m structure. Moreover, the multi-band nature of Pb$_2$Sb$_2$O$_7$ may also prevent the gap opening.

It has already been reported that the P-phase is stable in an Na-containing atmosphere, but the composition of the product becomes (Na,Pb)$_2$Sb$_2$O$_7$ and does not show a flat band^34^. Therefore, to synthesize P-phase Pb$_2$Sb$_2$O$_7$, some ingenuity is required. For example, Cai et al. pointed out that in some A$_2$B$_2$O$_7$ compounds, P- and W-phases compete, and a high-pressure P-phase exists in the stability field^38^. Therefore, high-pressure synthesis may be effective in certain cases. For example, Ca$_2$Sb$_2$O$_7$ and Cd$_2$Sb$_2$O$_7$ form the W-phase under ambient pressure, whereas the P-phase can be synthesized by high-pressure synthesis at 60–65 kbar^55^. Controlling other conditions, such as the atmosphere and temperature, may also be effective in obtaining a metastable P-phase. Regarding the high pressure, we also performed a first-principles calculation with a changing lattice volume for the cubic P-phase. Details of the calculations are described in Supplemental Material for details of the calculation, which includes Refs.^40–42^. We confirmed that applying pressure decreased the magnetic moment, but this effect was not major. After applying a pressure of 29.4 GPa, a magnetic moment of 0.14 $\mu _\text{B}$ remained. This also demonstrated the robustness of the flat band to the applied pressure.

Finally, there is a possibility that a unique Weyl point appears in P-phase Pb$_2$Sb$_2$O$_7$. A recent theoretical study has shown that when electron-doped Sn$_2$Nb$_2$O$_7$ becomes ferromagnetic, it has Weyl points owing to spin-orbit coupling^56^. These Weyl points may also be observed in Pb$_2$Sb$_2$O$_7$. However, because the signs of the effective spin-orbit coupling are opposite between Sn$_2$Nb$_2$O$_7$ and Pb$_2$Sb$_2$O$_7$, the Weyl points appear differently^48^. Moreover, Pb$_2$Sb$_2$O$_7$ does not become a Weyl semimetal because of the presence of a large Fermi surface derived from FB2. Furthermore, a small rhombohedral distortion may cause band crossing, as in Tl$_2$Nb$_2$O$_7$^52^, which may change the topological properties. Pb$_2$Sb$_2$O$_7$ will add a new variety to flat band systems^57,58^.

## Conclusions

In summary, we discovered that the top of the valence band of pyrochlore Pb$_2$Sb$_2$O$_7$ contained a quasi-flat band. This anomalous band dispersion originated from the geometric frustration lattice of the 3D Pb$_2$O$^{\prime }$ network. Unlike other previously proposed flat band compounds, in Pb$_2$Sb$_2$O$_7$, the carriers are self-doped by another band. In other words, the problem of carrier doping, which is the greatest hurdle for the metallization of flat band systems, is naturally solved. Although the problem of competition with the weberite phase remains, this material is a strong candidate for realizing doped flat band systems and magnetic materials that do not contain magnetic elements. This ferromagnetism is robust with respect to rhombohedral distortion and pressure application. Novel Weyl points attributed to spontaneous magnetization combined with spin-orbit coupling are also anticipated.

## Supplementary Information

Supplementary Information.

## Data Availability

Any data used to generate the results in this study can be obtained from the corresponding author upon reasonable request.
