# Supplementary material for: Flat band ferromagnetism in Pb2Sb2O7 via a self-doped mechanism
Source: Sci Rep. 2023 Mar 23;13:4743. doi: 10.1038/s41598-023-31917-w (PMC10036504; doi:10.1038/s41598-023-31917-w)
Supplement: Supplementary file 1 — Supplementary Information. [file 41598_2023_31917_MOESM1_ESM.pdf]

# Supplemental Material for "Flat Band Ferromagnetism in $Pb_2Sb_2O_7$ via a Self-Doped Mechanism"

I. Hase, Y. Higashi, and H. Eisaki
*National Institute of Advanced Industrial Science and Technology (AIST), Tsukuba Central 2, 1-1-1 Umezono, Tsukuba 305-8568, Japan*

K. Kawashima
*IMRA-JAPAN Material R&D Co. Ltd., 2-1 Asahi-machi, Kariya, Aichi 448-0032, Japan*
(Dated:)

In this supplemental material document, we present details regarding the band structure calculation and tight-binding analysis of $Pb_2Sb_2O_7$.

PACS numbers:

## I. DETAILS OF DENSITY-FUNCTIONAL THEORY CALCULATIONS

Based on the density-functional theory, we computed the electronic structure of $Pb_2Sb_2O_7$ based on first principles for pyrochlore and weberite structures. We used a full-potential augmented plane-wave scheme, and the exchange-correlation potential was constructed within the general gradient approximation [1]. For the computing code, we used the WIEN2k package [2]. The parameter $RK_{max}$ was set to 7.0. The $k$-point mesh was chosen so that the total number of mesh points in the first Brillouin zone was $\sim 1000$. The convergence of the atomic position was judged to be less than 1.0 mRy/a.u based on the working force acting on each atom. We optimized the lattice constants and atomic coordinates while retaining the space group for the pyrochlore structure to $Fd\bar{3}m$ (No. 227). Under this restriction, the only structural parameter to be optimized was $u$ in O($u$,1/8,1/8). The other atoms occupied the Wyckoff positions, as indicated in Table S1. These are denoted as "Origin B," where atom B occupies the origin (000). We obtained the value of the lattice parameter as $a = 10.8804$ Å, which is close to the experimental data [3]. Unfortunately, we could not determine an experimental value for $u$ from the literature, but our optimized value was $u = 0.31759$, which is close to the value of $u = 0.3151$ in pyrochlore oxide $Sn_2Nb_2O_7$. Note that the position of O in a previous paper [11] was denoted as "Origin A", where atom A occupied the origin (000). These two coordinate systems ("Origin A" and "Origin B") are related by the following relationship: $u_A + u_B = 3/4$.

After optimizing the crystal structure, we included spin-orbit interaction (SOI) using the second-variational approach. As explained in the main text, the valence band including the quasi-flat band primarily consists of Pb-$s$ and O′-$p$ orbitals. Therefore, the effect of the SOI is minimal with respect to the valence band width (although Pb is a heavy atom, the Pb-$s$ orbital does not possess angular momentum). For example, the energy splitting caused by the SOI indicated in Fig. 1(d) is only $\sim 35$ meV at point Γ.

### A. Pressure Effect

While optimizing the lattice constants in the above calculations, the pressure could be obtained as a function of the lattice constants by obtaining the total energy (with relaxed $u$) for each lattice constant. For example, $a = 10.5523$ and $a = 10.3301$ Å correspond to the pressures $P = 14.9$ and $P = 29.4$ GPa, respectively. We discovered that the flat band (FB) was extremely robust to changes in the lattice constant or pressure. Consequently, the ferromagnetic state was also robust to changes in the lattice constant or pressure. For example, we obtained the ferromagnetic moment as $M = 0.14\mu_B/$ $Pb_2Sb_2O_7$ for a pressure of $P = 29.4$ GPa. This robustness was also observed in other FB ferromagnetic systems, such as $Pb_2Ta_2O_6N$ [12].

### B. Rhombohedral Distortion

As stated in the main text, a high density of states (DOS) at $E_F$ can induce some instability, and lattice distortion may be one form of such instability. Previous reports [3, 4] have suggested that $Pb_2Sb_2O_7$ presents a rhombohedral distortion, but no precise information including that on space groups, lattice constants, and atomic positions has been reported. Therefore, we assumed a small rhombohedral distortion and investigated its effect on the electronic state. Under the assumed rhombohedral distortion, the Pb, Sb, and O sites were split into two species, resulting in a total of 22 atoms of seven species per primitive unit cell. In addition, the atomic positions of the two O sites and one O′

TABLE S1: Wyckoff atomic positions of pyrochlore $Pb_2Sb_2O_7$ for cubic and rhombohedral structures. Here, the origin denotes the Sb site (Origin B). For the rhombohedral structure, the coordinates represent the rhombohedral primitive unit vectors.

| Cubic, Fd$\bar{3}$m (No. 227) | | | Rhombohedral, R$\bar{3}$m (No. 166) | | |
|---|---|---|---|---|---|
| Pb | 16d | $(\frac{1}{2}\frac{1}{2}\frac{1}{2})$ | Pb1 | 3b | $(00\frac{1}{2})$ |
| | | | Pb2 | 9e | $(\frac{1}{2}00)$ |
| Sb | 16c | (000) | Sb1 | 3a | (000) |
| | | | Sb2 | 9d | $(\frac{1}{2}\frac{1}{2}\frac{1}{2})$ |
| O | 48f | $(u\frac{1}{8}\frac{1}{8})$ | O1 | 18h | $(\frac{1}{4}-u, u, u)$ |
| | | | O2 | 18h | $(\frac{3}{4}+u, \frac{3}{4}+u, \bar{u})$ |
| O′ | 8b | $(\frac{3}{8}\frac{3}{8}\frac{3}{8})$ | O′ | 6c | $(00v)$ |

site were relaxed owing to the reduced symmetry. The cubic and rhombohedral unit cells are depicted in Fig. S1, and the resulting space group and Wyckoff positions are summarized in Table S1.

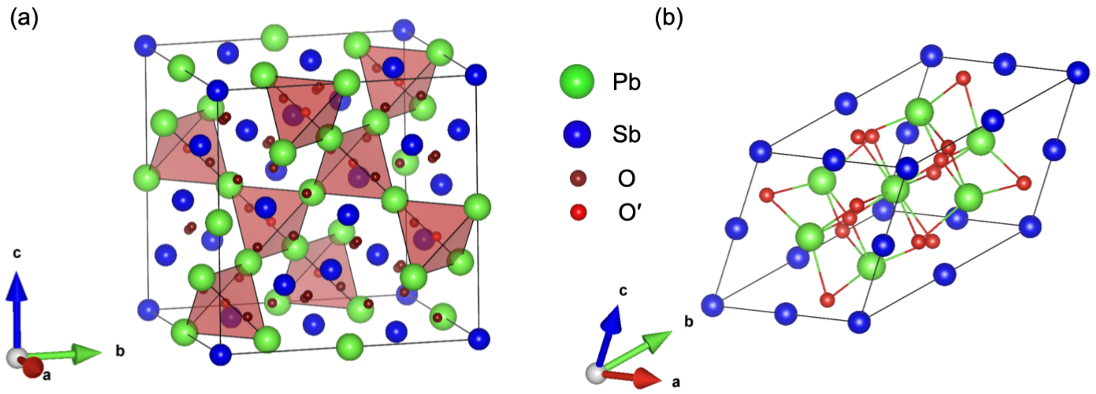


FIG. S1: Crystal structure of pyrochlore $Pb_2Sb_2O_7$ for the (a) conventional unit cell and (b) primitive unit cell. The rhombohedral unit cell is presented in (b), and it is the same as the cubic primitive unit cell. The illustration of the crystal structures was produced using VESTA [5]. The internal atomic positions in the primitive unit cell were generated using cif2cell [6].

We assumed a small rhombohedral distortion and investigated its effect on the electronic state. The mechanism of change in the Wyckoff position owing to this symmetry reduction has been evaluated previously [8–10]. We also noticed that the Wyckoff position 18h in the rhombohedral R$\bar{3}$m (No. 166) had two internal parameters [8]. Similarly, the Wyckoff position 6c in R$\bar{3}$m had one internal parameter, whereas the corresponding Wyckoff position 8b in Fd$\bar{3}$m had no internal parameters. Therefore, we must determine these internal parameters by minimizing the total energy of the system.

In a cubic crystal, there exists only one degree of freedom for the lattice constant, whereas a rhombohedral crystal has two degrees of freedom. Therefore, we gradually changed the lattice constant ratio $c/a$ while maintaining the

volume constant and optimized the atomic positions at each point. Consequently, the total energy was minimized when $c/a$ was 1 % smaller than that of the cubic crystal. Simultaneously, the total energy was stabilized by 0.03 eV/ $Pb_2Sb_2O_7$ compared to that of the cubic crystal. In the optimized structure, the O′ atom shifted considerably toward the Pb1 atom. The Pb-O′ bond lengths became $d$(Pb1-O′) = 2.23264 Å and $d$(Pb2-O′) = 2.39855 Å. The ratio of the bond lengths was $d$(Pb1-O′)/$d$(Pb2-O′) = 0.9308, implying a 7% difference. However, the $Sb_6O$ octahedra remained almost solid. The distribution of the bond lengths of (Sb1,Sb2)-(O1,O2) was limited between 2.54094 and 2.06321 Å, resulting in a minimum/maximum bond length ratio of 0.9960.

The obtained DOS and band dispersion are illustrated in Fig. 4 in the main text. Because the primitive unit cell is similar for the cubic and rhombohedral structures without a small distortion (1 % in this case), the shape of the first Brillouin zone is also the same. Therefore, we used the same $k$-point path to draw the energy dispersion in Fig. 4(b).

We noted that the FB split into two owing to the lowered symmetry. Nevertheless, ferromagnetism was observed even under this completely relaxed structure, and a magnetic moment of $M = 0.37\mu_B/$ $Pb_2Sb_2O_7$ was obtained.

### C. Weberite Structure

For the weberite structure (orthorhombic, space group I2cm, No. 46), we used experimental lattice parameters and atomic positions owing to the complexity of the crystal structure [7]. Nevertheless, we discovered that the weberite structure was more energetically favored than the pyrochlore structure. The obtained value of 0.549 eV per $Pb_2Sb_2O_7$, produced a lower bound for the stabilization energy of the weberite phase.

The DOS and energy dispersion of $Pb_2Sb_2O_7$ for the weberite structure are depicted in Fig. S2. For the weberite structure, we obtained a band gap of 2.08 eV, and no FB features were observed.

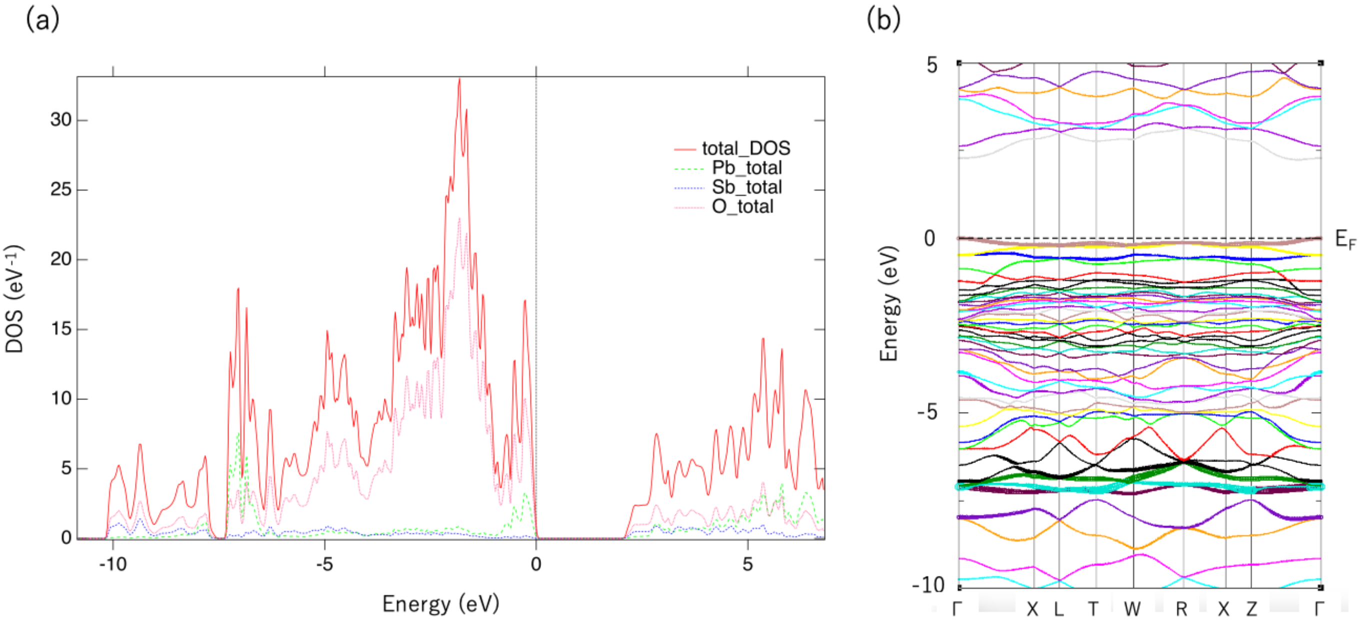


FIG. S2: (a) DOS curve and (b) energy dispersion of $Pb_2Sb_2O_7$ for the weberite structure.

## II. DETAILS OF THE TIGHT-BINDING CALCULATIONS

We used the calculation code Wannier90 [13]; this code constructs maximum localized Wannier functions (MLWFs) directly from the *ab initio* energy bands. For the MLWFs, we used localized orbitals with the same symmetry as the corresponding atomic orbitals, such as Pb-$s$ and Sb-$s$.

The MLWFs were constructed from the *ab initio* eigenvalues and eigenvectors on $8 \times 8 \times 8$ unshifted $k$-mesh points. The obtained tight-binding model was a four-orbital model including numerous transfer (or hopping) integrals, other than the nearest neighbor atoms. We perfectly reproduced the original bands using "Pb-$s$" MLWFs, as indicated in Fig. 1(b) in the main text (green curves, FB1), although the bottom of these bands (at point $\Gamma$ with an energy of $\sim -2.7$ eV) was entangled with the O-$p$ bands. Similarly, the bottom of the conduction bands could be perfectly described by "Sb-$s$" MLWFs, as indicated in Fig. 1(c) in the main text (green curves, FB2), although the top of these bands (at point $\Gamma$ with energy $\sim 3.0$ eV) was entangled with higher energy bands. The four largest parameters of these tight-binding models are listed in Tables I(a) and I(b). Readers may refer to the main text for further discussions.


[1] Perdew, J. P., Burke, K., & Ernzerhof, M., Phys. Rev. Lett. **77**, 3865 (1996).

[2] Blaha, P., Schwarz, K., Madsen, G. K. H., Kvashnicka, D. & Luitz, J. *WIEN2k, An Augmented Plane Wave plus Local Orbitals Program for Calculating Crystal Properties* (Vienna University of Technology, Vienna 2001).

[3] Brisse, F., Stewart, D. J., Seidl, V. & Knop, O. Can. J. Chem. **50**, 3648 (1972).

[4] Marchetti, A., Saniz, R., Krishnan, D., Rabbachin, L., Nuyts, G., De Meyer, S., Verbeeck, J., Janssens, K., Pelosi, C., Lamoen, D., Partoens, B. & De Wael, K. Chem. Mater. **32**, 2863 (2020).

[5] Momma, K. & Izumi, F., J. Appl. Crystallogr. **44**, 1272–1276 (2011).

[6] Bjorkman, T., Comput. Phys. Commun. **182**, 1183-1186 (2011).

[7] Ivanov, S. A. & Zavodnik, V. E., Sov. Phys. Crystallogr. **35**, 494 (1990).

[8] Aroyo, M. I. Ed.: *International Tables for Crystallography Volume A: Space-group Symmetry* (Sixth edition, Wiley, 2016)

[9] Aroyo, M. I., Perez-Mato, J. M., Capillas, C., Kroumova, E., Ivantchev, S., Madariaga, G., Kirov, A., & Wondratschek, H., Z. Kristallogr. Krist. **221**, 15-27, (2006).

[10] Aroyo, M. I., Perez-Mato, J. M., & Wondratschek, H., Acta Cryst. A **62**, 115-128 (2006).

[11] Hase, I., Yanagisawa, T., Aiura, Y. & Kawashima, Phys. Rev. Lett. **120**, 196401 (2018).

[12] Hase, I., Yanagisawa, & K. Kawashima, Nanomaterials **9**, 876 (2019).

[13] Mostofi, A. A., Yates, J. R., Lee, Y. -S., Souza, I., Vanderbilt, D. & Marzari, N., Comput. Phys. Commun. **178**, 685 (2008).
